# Crayfish Recognize the Faces of Fight Opponents

**DOI:** 10.1371/journal.pone.0001695

**Published:** 2008-02-27

**Authors:** Joanne Van der Velden, Ying Zheng, Blair W. Patullo, David L. Macmillan

**Affiliations:** Department of Zoology, University of Melbourne, Victoria, Australia; Freie Universitaet Berlin, Germany

## Abstract

The capacity to associate stimuli underlies many cognitive abilities, including recognition, in humans and other animals. Vertebrates process different categories of information separately and then reassemble the distilled information for unique identification, storage and recall. Invertebrates have fewer neural networks and fewer neural processing options so study of their behavior may reveal underlying mechanisms still not fully understood for any animal. Some invertebrates form complex social colonies and are capable of visual memory–bees and wasps, for example. This ability would not be predicted in species that interact in random pairs without strong social cohesion; for example, crayfish. They have chemical memory but the extent to which they remember visual features is unknown. Here we demonstrate that the crayfish *Cherax destructor* is capable of visual recognition of individuals. The simplicity of their interactions allowed us to examine the behavior and some characteristics of the visual features involved. We showed that facial features are learned during face-to-face fights, that highly variable cues are used, that the type of variability is important, and that the learning is context-dependent. We also tested whether it is possible to engineer false identifications and for animals to distinguish between twin opponents.

## Introduction

Visual recognition is poorly understood throughout the animal kingdom [1]–[4]. The occurrence and use of cognitive processes such as recognition by invertebrate animals is therefore significant because of its implications for our understanding of the evolution and use of learning and memory processes. Humans use complex recognition to organize their lives but it has been postulated that the underlining processes may be relatively simple [5], [6] and that commonalities might be testable across disciplines and species [7].

Invertebrates are valuable for studying visual processes because they permit us to pose questions that cannot easily be tested in vertebrates with their more complex behavior and neural processing power. Some recognition processes occur in invertebrates, for example, in bees and wasps which live in colonies where the benefit of recognizing fellow nest mates contributes to successful function of the colony [3], [8]. Other invertebrates, including some decapod crustaceans, do not exhibit strong social cohesion, but are highly visual; it is not known whether they have visual recognition. That is, some crayfish have sparse natural distributions [9] and individuals have short fights and then separate [10]. They can form hierarchies in research laboratories [11], [12], but the associations are weak and relative positions change depending on experience in previous contests [13] so one might not predict strong selection for visual recognition of other individuals.

There is evidence that visual cues are important to decapods, with most studies using artificial manipulations to demonstrate recognition. Aspects of aggressive behavior in interactions between individuals are affected by varying the size of natural white markings on the chelae of *Calcinus laevimanus* with white paint [14], manipulating body patterns on *Calcinus tibicen* [15] and attaching identity tags to the carapace of *Potamon fluviatile* [16]. Fiddler crabs can distinguish between species and mates when natural patterns are modified with paint and they will also approach unpainted unfamiliar conspecifics in preference to familiar ones [17]. The level of illumination also affects behavioral interactions with aggressive acts lasting longer and being more intense in dim light as opposed to bright light [18]. There is also physiological evidence that the most advanced colour sensitivity described in the invertebrates occurs in stomatopods [19], [20].

Australian crayfish, *Cherax destructor*, occupy environments ranging from clear streams, to turbid ponds and they are also active in daylight and at night and could therefore benefit from visual recognition ability. We investigated whether individuals can visually recognize fight opponents.

## Results

**Figure 1:**
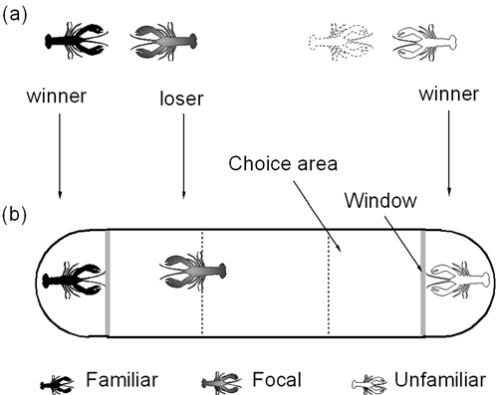
Test paradigm for crayfish visual recognition. (*a*) Fights between size-matched crayfish to familiarize opponents. These fights occurred, one pair at a time, in the central area of the tank shown. The tank was cleaned between encounters. (*b*) Winners and losers were transferred to the test arena with a pen and choice area at each end. The focal losing crayfish could spend time in any of the three areas, two of which would indicate preference for proximity to a specific animal. The figure shows a focal crayfish visiting the familiar animal from the previous encounter. Features, for example facial width and color, were varied between the stimulus animals in the pens. The window prevents chemical and mechanical cues from passing but does not interfere with vision.

**Figure 2:**
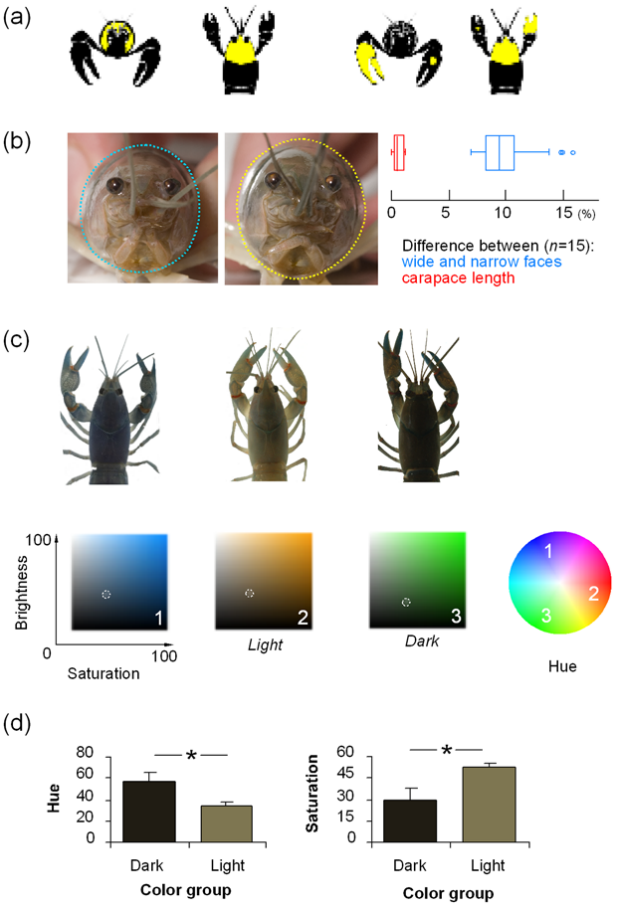
Manipulating visual clues. Testing artificial paint patches (*a*), facial width (*b*) and color of the facial region (*c–d*) for a recognition response in *C. destructor*. (*a*) Images of crayfish painted in the face region (left) and chelae (right) from the front and top perspectives. Two paint experiments are shown on the chelae images: the chelae experiment and the small paint patch (control 2). Chelae paint was applied to both chelae (only one shown) and to the dorsal and ventral surfaces (only dorsal shown). (*b*) A narrow- and a wide-faced crayfish were placed at either end of the choice arena. Left panel shows an example of a “narrow” and “wide” face (8% wider, scale: square face panels 2×2 cm). The box plots (right panel) summarize the difference in facial width (blue) between the narrow and wide stimulus crayfish in one experiment and the small difference between their carapace lengths (red). (*c*) Different colored crayfish were placed at the ends of the choice arena. To make the distinction between crayfish, three color elements were measured: hue (*color*, as depicted by a color wheel-shown left), saturation (*intensity*) and brightness (*lightness or darkness*). The three partial crayfish pictured show the range of color variation we measured in *C. destructor*. The color swatches (1 to 3) beneath each crayfish image represent the saturation and brightness of color on a scale of 0–100% that were measured for that crayfish image; the hue for each image is shown on the color wheel to the right. (*d*) When color was varied, the stimulus crayfish were grouped into a “light” and a “dark” color, judged by human eye and confirmed by analysis. Crayfish images 2 and 3 in (b) represent the means of the hue and saturation of the light and dark groups. Brightness was not used to judge crayfish different because this is dependant on the available light. Graphs show difference between the stimulus crayfish for hue and saturation (mean±s.e.m, *significantly different *t* test p<0.05 and also visibly different by human eye as confirmed by naïve observers asked to group the crayfish images by color). Images were photographed under standardized conditions in a studio.

To test whether *C. destrtuctor* can visually recognize an opponent and determine if some parts of the body are more valuable for recognition than others, first we exploited evidence that addition of artificial color patches and other greebles [21] changes the way crustaceans behave toward conspecifics [14]–[17]. Preliminary experiments suggested that *C. destructor* might also be capable of this form of recognition. Because *C. destructor* face their opponents most of the time during agonistic encounters we reasoned that anterior regions of the body might be important for recognition and painted patches on their faces (Yellow correction fluid, Papermate). We defined the “face” as the region of the crayfish anterior to the cephalic groove (the cephalothorax) because some of this area is always in view during the head-on encounters that characterise fights in this species. We use the term “face” also because this part of the animal matches the human perception of “face” so it is readily understood. We also painted patches of the same size on the claws of another group of crayfish. Painted animals were matched against control-painted crayfish in a familiarization encounter in an arena (Fig. 1, 2a).

After the encounter, unpainted losers were selected as focal crayfish to control for social status, placed in the arena and tested for recognition ability (Fig. 1). We placed the original familiar winner randomly in one pen and a size-matched, unpainted, unfamiliar winner in the other.

When the paint patch was applied to the facial region the focal crayfish demonstrated recognition by spending more time in the end with the familiar, painted individual (mean±s.e.m.: familiar end 273±21 s, unfamiliar end 199±18 s; Wilcoxon n = 15, z = −2.073, p = 0.038). When the patch was applied to the claws the focal crayfish showed no preference for either end (familiar end 229±15 s, unfamiliar end 249±15 s; Wilcoxon n = 15, z = 0.568, p = 0.570).

Control experiments showed that *C. destructor* have no preference for crayfish with a yellow patch on their cephalothorax if they have not encountered them before (familiar end 257±24 s, unfamiliar end 225±21 s; Wilcoxon n = 15, z = 0.682, p = 0.496), that the paint did not affect behavior of the focal crayfish in this design (familiar end 244±14 s, unfamiliar end 236±21 s; Wilcoxon n = 15, z = 0.568, p = 0.570), that focal crayfish do not change their behavior toward a familiar animal when it is visually alike the unfamiliar-i.e. “twins” (familiar end 245±16 s, unfamiliar end 218±23 s; Wilcoxon n = 15, z = −0.398, p = 0.691), and that there was no bias in the experimental apparatus (familiar end 227±15 s, unfamiliar end 223±23 s; Wilcoxon n = 15, z = −0.170, p = 0.865).

This demonstrates that under the conditions of this test, *C. destructor* can use patches of paint on the head for recognition but that they do not use those on the claws in the same way. While it is possible that the crayfish recognized the claw-marked individuals but responded differently, the simplest explanation is that they did not pay sufficient attention to the claw markings to permit them to later identify the marked individual. Either option suggests that the face is more important for recognition when this is established during physical encounters that involve substantial amounts of face-to-face interaction as in our test case.

The color marking experiments suggested that *C. destructor* might find the head and facial region of particular interest for recognition, so we investigated whether some natural features in this area have sufficient variation to be used in visual recognition. Features used for recognition in size-matched crayfish might be expected to show more variation than ones that are not used because combinations of such factors would provide more distinctive sets of cues. We therefore examined two facial features in crayfish of the same size and found that those potential visual cues were not correlated with body size (length) or each other, suggesting that facial features could be used in recognition.

We chose facial width as a character and classified crayfish of the same size into “narrow-” and “wide-faced” individuals. The narrow and wide faces were selected by visual inspection of the animals and measured with callipers to quantify the difference (dotted lines on photos in Fig. 2b indicate the width). The difference in width was about 10% (Fig. 2b box plot); a similar disparity in size is known to influence other crayfish behaviors [22] so we reasoned this should also be sufficient to be recognized. We then ran familiarization encounters as before and tested for recognition by placing the focal animals in the arena and familiar or unfamiliar winners of different facial width category in the pens. When focal crayfish were given a choice between a familiar winner and an unfamiliar winner with a different facial width, they chose to spend more time in the proximity of the familiar animal (familiar end 266±21 s, unfamiliar end 189±15 s; paired t-test n = 15, t = 2.337, p = 0.035). This demonstrates that following a fight encounter, *C. destructor* can recognize a familiar individual and that facial width, or features associated with it, is involved in that process.

Since it is likely that other cues are also used, we tested whether color (hue and saturation), which also varies in *C. destructor* of the same carapace length, might be involved (Fig. 2c–d). When we repeated our experiment selecting for color instead of width, the focal crayfish showed no preference for the familiar or unfamiliar individual (familiar end 269±14 s, unfamiliar end 224±10 s; paired t-test n = 15, t = 1.914, p = 0.076). In this case, highly variable elements of a potentially significant identifier did not predict recognition and we conclude that high variability alone does not indicate that a feature will be used for recognition in a particular situation. The result could be interpreted as a strong trend and one of possible biological significance, but further testing in different behavioral situations and with further dissection of the components of color would be required to establish this conclusion.

*C. destructor* can recognize familiar crayfish based on at least one factor that varies independently of carapace length. If a feature such as facial width alone is sufficiently important for recognition, we reasoned that it might be possible to trick crayfish into making false identifications by using non-familiar animals with their facial widths closely matched to those of the crayfish encountered during the agonistic learning encounter. To do this we matched the familiar winner with a “pseudo-familiar winner” and substituted the latter at the choice stage of the experiment i.e. one test pen contained the pseudo-familiar winner, the other contained a non-familiar winner with different facial width to the familiar, as before. The focal crayfish behaved as if they had not seen either stimulus animal before–their choice of end was not significantly different from the control condition (familiar end 252±26 s, unfamiliar end 268±28 s; paired t-test n = 15, t = 0.300, p = 0.496)-and we conclude that facial width alone was not sufficient for a recognition response where there is no previous physical encounter. This suggests other visual cues are also involved.

**Figure 3:**
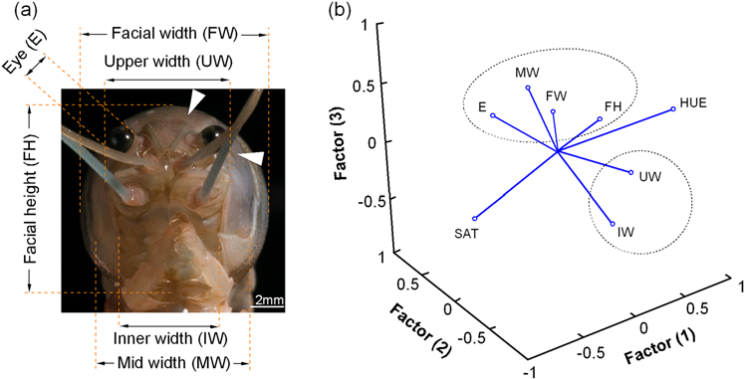
The face of *C. destructor*. (*a*) Photo of the features analyzed on *C. destructor's* face. Color hue and saturation were taken from the mean of 5 points (white triangles are 2 example locations). (*b*) PCA factor analysis of 8 facial features of *C. destructor*. Two clusters are identified by dotted circles and the color variables (HUE–color, SAT–saturation) occur at the extremes of the plot and outside these clusters. Facial width (FW) and color (SAT, HUE) do not plot together indicating that their variation is different.

Our contrasting results between width and color indicate that high variability alone is not sufficient to predict that a cue will be used in recognition. It is possible that the nature of the variability is important. To test this we measured eight facial characters (Fig. 3a) in a group of size-matched individuals (*n* = 17) and performed a correlation analysis for each. All showed considerable variation (Pearson correlation coefficients ∼0.1–0.5, Fig. S1). To determine whether the group was homogeneous or whether different types of variation were represented, we performed a Principle Components Analysis (PCA). This separated the features into clusters based on their variability and, notably, facial width and color were widely separated (Fig. 3b). This suggests that either cues with particular variation patterns are favoured for recognition by *C. destructor* or that cues drawn from clusters with different variation patterns are used. In either case, this is evidence that the pattern of variation can be an influential factor. This is consistent with the recent prediction that arthropods would necessarily be limited in the number of cues they could use for recognition because of the processing limitations of their nervous systems [3].

How animals perceive and interact with objects depends on experience [23]. If this applies during visual recognition, the response of *C. destructor* could be different depending on the context of the learning. Our experiments demonstrating recognition were all conducted on crayfish drawn from communal tanks prior to the experiment. We therefore repeated the facial width and color recognition experiments on crayfish that had been isolated for 2 weeks prior to the test. In contrast to the earlier result, crayfish showed no preference for familiar opponents (width: n = 15, paired t-test t = 0.408, p = 0.689; color: n = 15, paired t-test t = 0.055, p = 0.957) even though the isolated crayfish had longer fights (ANOVA df = 1, MS = 625164.8, F = 10.717, p = 0.001). That is, the previous social history had an effect either on *C. destructor's* ability to recognize antagonists from previous encounters or their behavioral response to recognition altered.

## Discussion

Crayfish recognized each other using natural facial features learned during a fight. Our experiments suggest that the opponent's facial region is committed to memory for at least 24 hours. This is a similar time period over which *C. destructor* remember topography of their environment [24]. Memory of a previous opponent has been shown to influence aggressive behaviour for up to two weeks in *C. destructor* [25] so it is possible that the learning of an opponent by visual recognition persists longer than 1 day. The memory may also be affected by the level of dominance of the focal crayfish as there is evidence that subordinates of the crab *Chasmagnathus granulatus* have better memory retention than dominants [26].

There is evidence to support both suggestions from the isolation experiments: that the isolation affected the ability of *C. destructor* to recognize conspecifics or it altered the behavioral response. *C. destructor's* ability to recognize may have changed because the memory ability of decapods can differ between subordinates and dominants [26]. On the other hand, *C. destructor* may have still been able to recognize the opponents, but changed their behavioral response, because isolation is known to affect behavioral outcomes in aggressive contests [25] and in our experiments it also changed behavior-isolated crayfish fought for longer than communal ones. Knowledge of the effect of social isolation on crustaceans is limited. It is known that isolated individuals of *Pargurus samuelis* win more encounters [27] and recent evidence suggests isolation also affects aggressive behavior in *C. destructor* [25]. Our outcome indicates that the absence of social experiences (isolation) can also affect the behavior of crayfish towards familiar individuals.

The behavioral response in which crayfish prefer to remain close to a familiar animal has been demonstrated before in *C. destructor* using chemical cues [10]. It is also evident in shrimp species [28], [29] and in a range of vertebrate species [30], [31]. This behavior, known as the “dear enemy” phenomenon [*sensu* 32], could reduce the energetic cost and physical damage from high intensity fights that occur between unfamiliar crayfish [10]. There is also evidence that decisions by an individual to associate with familiar conspecifics confers other advantages because it allows the individual to direct behaviors to other situations, e.g. predator avoidance or feeding [33]. This supports the recent suggestion that solitary species, of which *C. destructor* is one, will adopt the strategy of approaching and spending more time with familiar individuals, compared with higher aggression toward familiars that is found in species that are in close social contact [34].

Colonial wasps and bees are the only other invertebrates known to be capable of visual recognition of familiar conspecifics [1], [8], [35], [36]. Our finding is unexpected in a species that does not have a strict social life like a colonial insect. Laboratory experiments show that crayfish can recognize status and identity using chemical cues [10], [37]–[39] and that they form hierarchies [11]. In the wild, some decapod species share shelters and live in small communal groups or exhibit gregarious behaviours [40]–[44]. They also form pair bonds [45] and recognition of chemical cues can be used in this relationship (e.g. [46], [47]). Detto and colleagues demonstrated visual recognition between crab mates using tethers and painted markings [17]; our observations in unrestrained *C. destructor* also suggest this is possible. We are not aware of strict pair bonds or group living in *C. destructor*, nor any structure like the colonies of bees and wasps, however a neighbourhood of individuals could exist in the wild, particularly for juveniles when they are shed from the mother until they disperse. There is evidence of this in juvenile *C. destructor* which form groups in the laboratory [48]. At this time, visual recognition would assist in maintaining relationships and territorial boundaries. In any case, visual recognition in *C. destructor* could be used in concert with chemical clues to increase the distance over which recognition could occur during daylight hours (e.g. chemical clues described for recognition in crayfish and other crustaceans [10], [38], [39], [49]–[52]).

The outcome strengthens recent evidence that crustaceans have true individual recognition [53], a controversial topic for the past 30 years [49], [54]–[56]. We conclude that familiar recognition underpins the behavior however, given the natural variation in features described, the likelihood that crayfish recognize multiple individuals from a set of visual cues is a possibility. Regardless of the type of individual recognition demonstrated, the benefits for life strategies are likely to be the same.

There is an emerging view that experimentation into behavior based on cognitive abilities will be advanced by greater attention to distributed theories of cognition and those that allow for the generation of testable hypotheses across disciplines [7]. *C. destructor's* ability to learn facial features in biologically relevant contexts, is a promising new candidate for such testing. Cues with high variability are involved, but unexpectedly, this alone does not predict that they will be used. This evidence calls into question whether recognition evolved in invertebrates to be further developed in vertebrates or whether two different processes are involved.

## Materials and Methods

### Studying recognition in crayfish

Most crayfish species are naturally aggressive towards conspecifics of both genders, whether or not specific resources are at issue when they meet [11]. All experiments were based on the longstanding finding in *Cherax destructor*, and many other crayfish, that all agonistic contests between two non-moulting, non-sexually-active individuals are predictably won by the crayfish that has at least a 5% longer carapace, regardless of gender [22]. Human observers cannot predict the winner where the difference is less and the contests are more protracted than when it is greater [22]. These facts ensure that when crayfish are size-matched they will spend time in extended, face-to-face, agonistic encounters before one becomes the winner (dominant animal) and the other becomes the loser (subordinate animal).

Specimens of *C. destructor* (Clark; 25–35 mm carapace length, both sexes) were obtained from commercial suppliers in New South Wales, Australia and maintained in husbandry tanks prior to testing (18°C, 12h/12h light/dark). Crayfish were only used once and individuals in a given trial were taken from different holding tanks. They were maintained in holding tanks for two weeks, either isolated or communally depending on the experiment.

### Apparatus

The experimental apparatus was a perspex aquarium separated into three sections, a central arena and two curved holding pens, by two transparent, non-porous perspex partitions (Fig. 1). The curved pens induced stimulus crayfish to face the center of the tank more than other designs (e.g triangular or rectangular ends; Y. Zheng unpublished data). Thus, when animals saw each other at the tank ends, there was considerable time in face-to-face view because the focal animals approached the ends of the tank head-on as they moved around the narrow space. This was important because crayfish spend most of their fight time head-on, so visual recognition could relate to this region. The sections were filled with tap water to a depth of 10 cm. Stimulus crayfish in the pens were isolated mechanically and chemically from those in the central arena but they could see each other. This was modified from a previous apparatus used to test crayfish for recognition following a fight [10]. The tank was positioned in a temperature controlled room with fluorescent illumination.

### Familiarization prior to testing procedure

Agonistic encounters to familiarize crayfish with each other were conducted in the central arena (Fig. 1a). Two crayfish were placed in opaque cylinders in the arena and allowed to settle for 2 minutes. The cylinders were removed so that the animals could interact. The interaction was scored according to criteria adapted from previous studies on crayfish and lobsters, in our laboratory and others [10], [11], [57]. The winner and loser were kept in separate, isolated holding tanks until use in subsequent recognition tests 24 h later. The apparatus was washed thoroughly between encounters to remove chemical traces [10].

### Recognition test procedure

To test for recognition behavior, we made video recordings of the focal crayfish in the central arena which was divided for scoring using boundary markers, into three equal, imaginary areas: a central zone and two end zones (Fig. 1b). To conduct a test, size-matched stimulus crayfish were placed in the pens at the ends and the focal crayfish placed in an opaque cylinder in the middle of the central arena to settle for 2 minutes. When the cylinder was removed, the focal animal moved around the arena. To allow for the effects of removal and the novelty of the arena, the first 2 minutes of activity were not scored. Following this, the crayfish was tracked for 10 minutes. It was free to choose where it spent its time and could indicate this by a preference for one or other of the stimulus crayfish or for the central zone. For consistency, a focal crayfish was deemed to have entered a zone when its rostrum crossed the boundary [10]. The pen containing the familiar crayfish was randomly varied to eliminate the possibility of positional effects. The recorded video footage was scored double blind so that the scorer had no knowledge of whether a familiar crayfish was present or of which pen it occupied. Systat v11 was used to analyze the total time spent in each end zone and α set at 0.05 [58].

### Synopsis of experiments

The choice animals at either end of the arena given to the focal crayfish for the recognition test are listed below (see Fig. 2a for images of paint locations). Combinations are the Familiar winner *vs* Unfamiliar winner and are similar in all respects except for the variations in the list.

Cephalothorax paint *vs* No paint

*Head region recognition-chephalothorax painted*

Chelae paint *vs* No paint

*Claws recognition-dactylus and propodus painted*

Control 1: Cephalothorax paint *vs* No paint

*Initial preference for paint–cephalothorax painted but no prior fight, only choice test*

Control 2: Small patch *vs* No paint

*Paint effect on behaviour–small paint patch applied to claw*

Control 3: No paint *vs* No paint

*Distinguishing twins–natural features visually matched by eye*

Control 4: Empty holding pen *vs* Empty holding pen

*Bias in the apparatus–no choice crayfish in the pens*

Narrow face *vs* Wide face

*Natural feature width–measured with callipers to ∼10% difference*

Light colored face *vs* Dark colored face

*Natural feature color–quantified hue and saturation from digital images*

“Pseudo-familiar” *vs* Unfamiliar

*Tricking the focal–a pseudo familiar with visually matched natural features to the real familiar*

Narrow face *vs* Wide face

*Isolation and facial width–animals isolated prior to the fight*

Light colored face *vs* Dark colored face

*Isolation and color–animals isolated prior to the fight*

### Note on color experiments

Crayfish were initially grouped into light and dark animals and then color was measured from digital images. The photographs were taken with a Nikon D2 digital SLR and Nikon 60 mm 1:2.8D lens set at constant aperture (F25) in controlled lighting (dark room with Monobloc ProSeries 1000 flash). File storage was RAW format on the camera which was converted to uncompressed 16-bit TIFF for viewing in Photoshop (Adobe, CS2). The images were standardized against a reference grid image with a grey and color swatch which was also photographed. The eye dropper tool was then used to measure small areas of colour, 5×5 pixels. Six measurements were taken, one from each square of a grid template that was held on the computer monitor, superimposed over the crayfish cephalothorax. This equipment setup and file protocol minimised loss of color information in the images [59]. Even with this care in image analysis, there could be information lost [59], e.g. we did not measure UV or polarized light. Nonetheless, a similar method of analysis to ours has been applied in previous studies and provides an indicator of some of the elements of the variation in cephalothorax color e.g. [60].

Could the focal crayfish see the different colors of the choice animals? We used natural variation of the color we saw on the cephalothorax and quantified this as an index of appearance. Crayfish are not known to have color vision but they have visual pigment sensitive to wavelengths of light near those reported in our study (peak sensitivity ∼570 nm, [61]). The “light” and “dark” color categories used in our experiments corresponded approximately to 620–750 nm and 570–590 nm (converted from the hue measurements we recorded, corresponding to red and yellow hues). It may have been possible therefore, that the focal crayfish were able to view one of the choice animal's appearances in more detail than the other because one of the color groups used was closer to the peak visual sensitivity. Also, if crayfish viewed the choice animals from angles other than that taken for the analysis, a different color to what we report may have been seen, but this would be infrequent given the apparatus design maximised the time animals faced one another.

Bearing in mind that it is difficult to control and measure all those variables for color in a live situation between two fighting animals, we left color in the analysis. However, we also ran the PCA model again without the hue and saturation variables. The result was similar: variables clustered into at least 2 groups on the factor plot, and the 3 PCA components explained 81% of the total variance (unpublished data). The main conclusion from the PCA remains the same: there were features that varied, but because they separated on the factor plot, they do not vary equally.

## Supporting Information

Figure S1 Principle Components Analysis. Scatterplots of the variation in visual cues measured in *C. destructor*.
(0.20 MB PDF)
