# Supplementary material for: Crayfish Recognize the Faces of Fight Opponents
Source: PLoS One. 2008 Feb 27;3(2):e1695. doi: 10.1371/journal.pone.0001695 (PMC2257977; doi:10.1371/journal.pone.0001695)
Supplement: Figure S1 — Principle Components Analysis. Scatterplots of the variation in visual cues measured in C. destructor. (0.20 MB PDF) [file pone.0001695.s001.pdf]

Supporting information figure 1

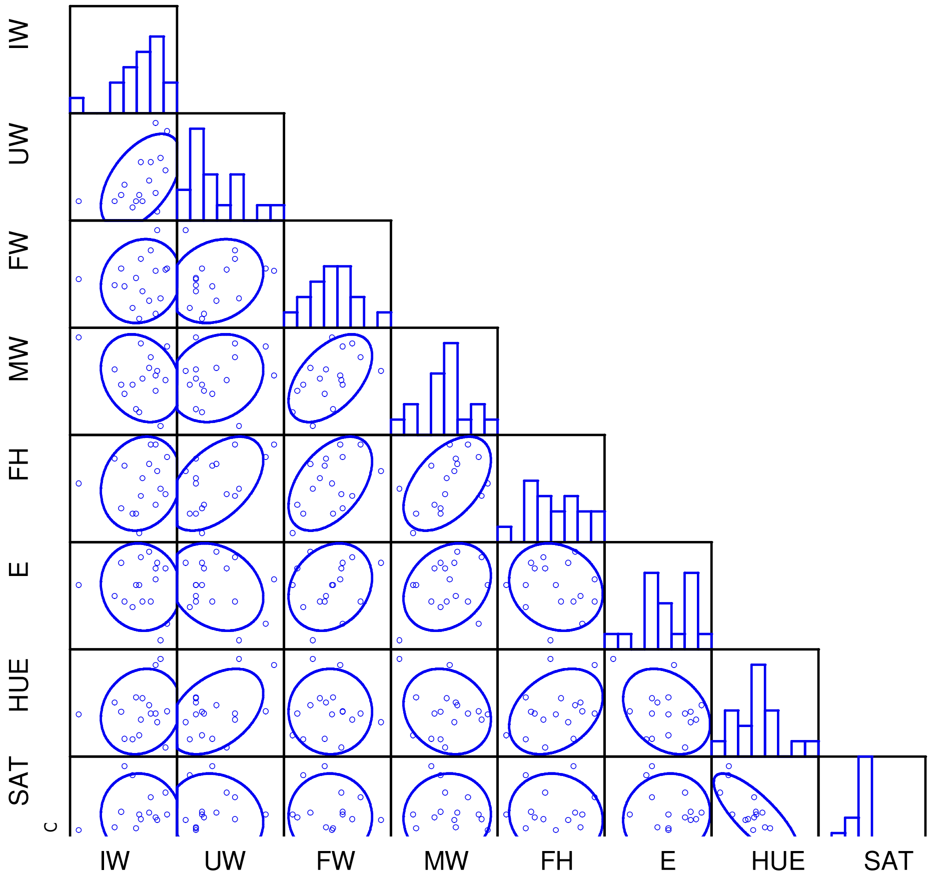

Table of Pearson Correlation Coefficients

	IW	UW	FW	MW	FH	E	HUE	SAT
IW	1.000	.	.	.	.	.	.	.
UW	0.518	1.000	.	.	.	.	.	.
FW	0.121	0.210	1.000	.	.	.	.	.
MW	-0.234	0.199	0.512	1.000	.	.	.	.
FH	0.148	0.503	0.473	0.501	1.000	.	.	.
E	-0.073	-0.243	0.214	0.263	-0.164	1.000	.	.
HUE	0.152	0.421	-0.004	-0.183	0.342	-0.301	1.000	.
SAT	-0.048	-0.144	-0.013	0.050	-0.094	0.086	-0.794	1.000

Abbreviations
IW inner width, **UW** upper width, **FW** facial width, **MW** mid width, **FH** facial height, **E** eye, **HUE** hue, **SAT** saturation.
